# Supplementary material for: LoDEI: a robust and sensitive tool to detect transcriptome-wide differential A-to-I editing in RNA-seq data
Source: Nat Commun. 2024 Oct 23;15:9121. doi: 10.1038/s41467-024-53298-y (PMC11500352; doi:10.1038/s41467-024-53298-y)
Supplement: Supplementary file 1 — Supplementary Information [file 41467_2024_53298_MOESM1_ESM.pdf]

Supplementary Information for

LoDEI: a robust and sensitive tool to detect transcriptome-wide differential A-to-I editing in RNA-seq data

Phillipp Torkler, Marina Sauer, Uwe Schwartz, Selim Corbacioglu, Gunhild Sommer, Tilman Heise

Contents

List of Supplementary Figures ii

List of Supplementary Tables ii

1 C. elegans analysis 8

2 Comparison of single samples 9

3 Implications of a window-based differential A-to-I editing calculation 11

4 Supplementary Tables 13

References 15

List of Supplementary Figures

1 Manual validation of differential A-to-I editing as detected by LoDEI in MYCN-amp cells 1
2 Manual validation of differential A-to-I editing as detected by LoDEI in *sncRNA7SL* cells 1
3 Alu Editing Index 2
4 Distributions of the average number of A-to-I positions per window 2
5 Upset-like plot for the comparison of detected windows and sites 3
6 Distributions of $\delta^{A \to G}$ values in the ADAR KD dataset 3
7 Impact of the window size on the number of detected windows 4
8 Empirical q values and absolute number of detected windows 5
9 Manual validation of differential A-to-I editing as detected by LoDEI in the RO60 KO dataset 6
10 Manual validation of differential A-to-I editing as detected by LoDEI in the MYCN dataset 7
11 Overlap of genomic positions of LoDEI windows with different window sizes 7
12 Observed signal differences and empirically derived q values 8
13 Differential A-to-I site performance comparison in *C. elegans* datasets . . 9
14 Distributions of the average number of A-to-I positions per window in the *C. elegans* datasets 9
15 Pairwise comparison naming scheme 10
16 Jaccard indices of detected differential A-to-I editing obtained from single sample comparisons by LoDEI 11
17 Implications of a window-based differential A-to-I editing detection 12

List of Supplementary Tables

1 LoDEI results for all possible mismatches for all analyzed datasets 13
2 DESeq2 results for genes of the ADAR family 14

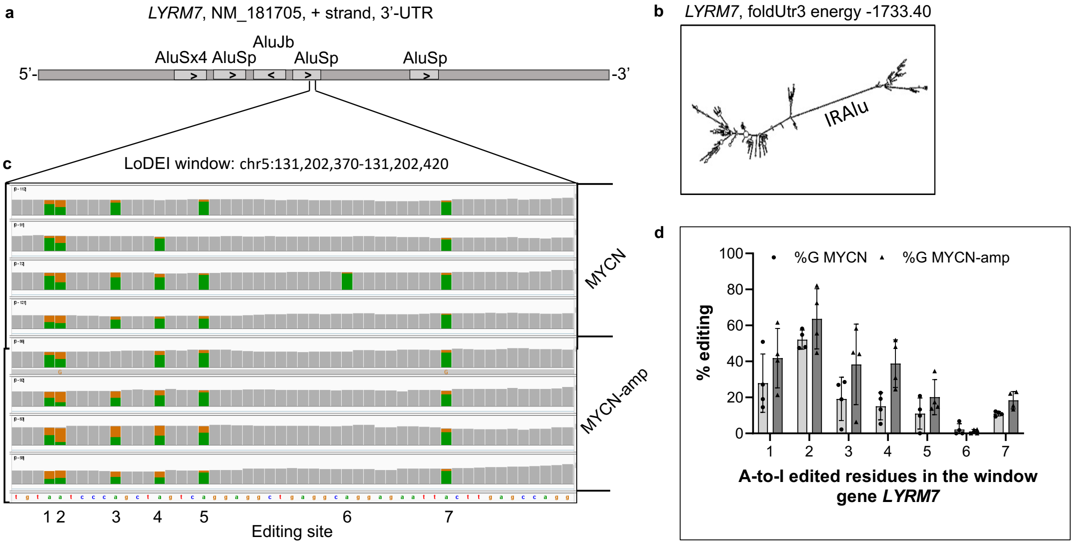

Supplementary Figure 1: Manual validation of differential A-to-I editing as detected by LoDEI. **a**, Scheme of the LYRM7 3'-UTR with five Alu elements. **b**, Predicted folding of the 3'-UTR and a long dsRNA IRAlu. **c**, IGV browser screen-shot of a LoDEI window with A-to-I editing sites (green A, orange G). **d**, Quantification of A-to-I editing shows a trend to more editing in MYCN-amp cells (n=8)

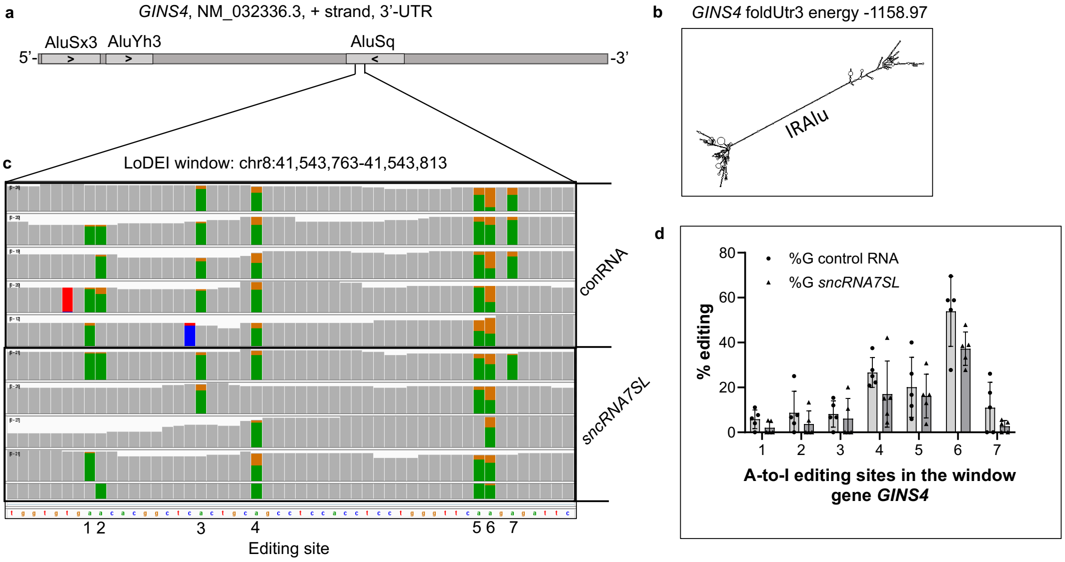

Supplementary Figure 2: Manual validation of differential A-to-I editing as detected by LoDEI. **a**, Scheme of the GINS4 3'-UTR with three Alu elements. **b**, Predicted folding of the 3'-UTR and the long dsRNA IRAlu. **c**, IGV screen-shot of a LoDEI window with A-to-I editing sites (green A, orange G). **d**, Quantification of A-to-I editing shows a trend to less editing in *sncRNA7SL* OE cells (n=10).

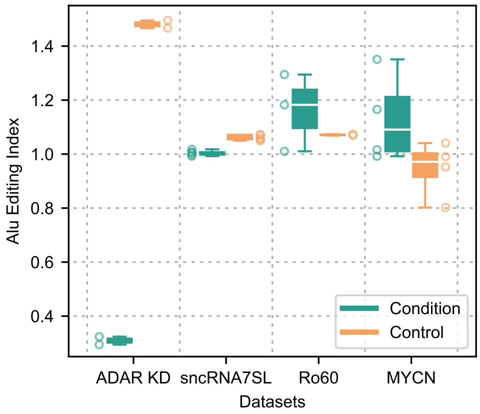

Supplementary Figure 3: Alu Editing Index. Individual AEI values for each sample are shown for the condition and control sets for all datasets. Boxplots are computed from individual AEI values. Condition refers to ADAR1 KD, *sncRNA7SL* OE, RO60 KO and MYCN-amp samples.

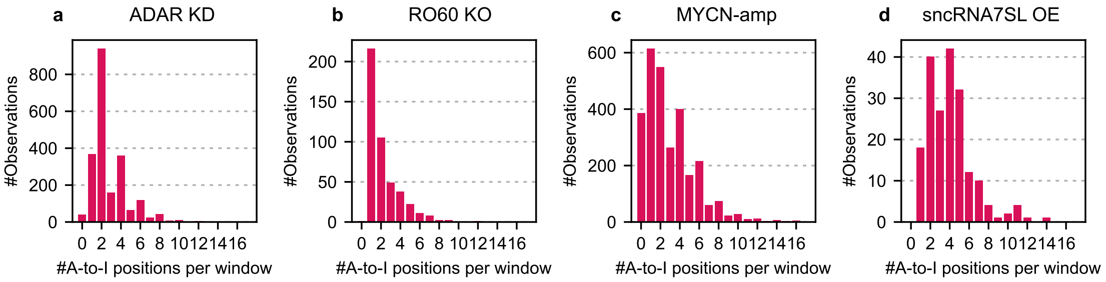

Supplementary Figure 4: Distributions of the average number of A-to-I positions per window. Shown are the average number of A-to-I sites within detected differential A-to-I windows by LoDEI in the ADAR KD (a), RO60 KO (b), MYCN-amp (c) and *sncRNA7SL* (d) datasets.

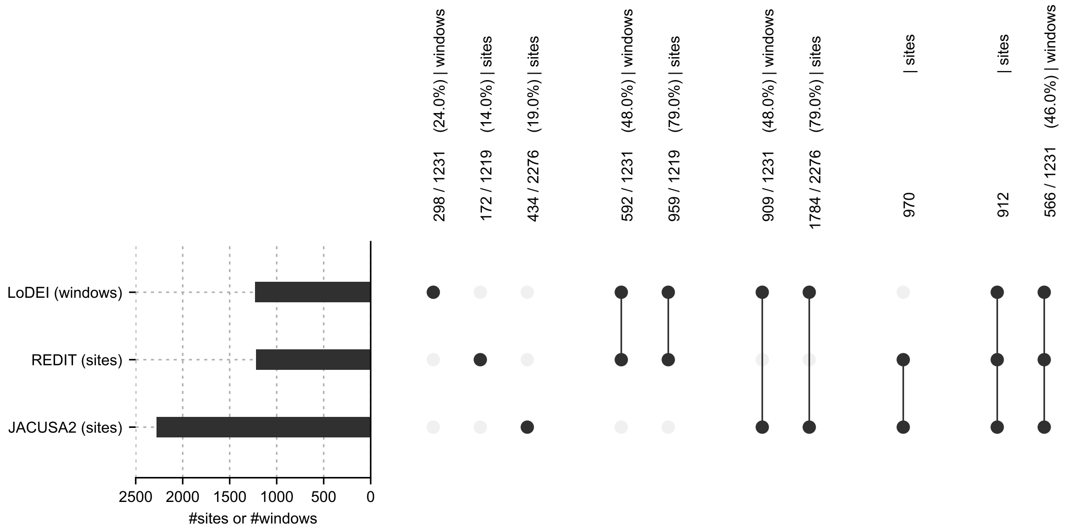

Supplementary Figure 5: UpSet-like plot for the comparison of detected windows and sites. UpSet plots visualize the intersections and relationships between sets [2]. The total number of detected differential A-to-I windows (LoDEI) or differential A-to-I sites (REDIT, JACUSA2) in the ADAR KD dataset for a q value threshold ≤ 0.05 are shown in the horizontal bar plot on the left. Intersections are visualized by dots. A single dot represents differential A-to-I windows or sites that are unique for each of the methods. Connected dots symbolize intersection of sets. Note, since LoDEI detects windows and not single sites, the intersections between LoDEI and any other tool must be made from the perspective of windows and sites. Thus, two intersections are given per comparison.

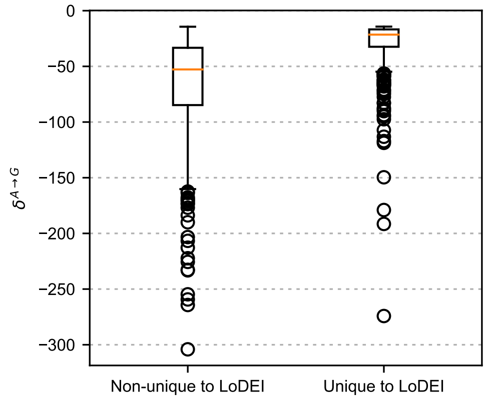

Supplementary Figure 6: Distributions of $\delta^{A\to G}$ values in the ADAR KD dataset. The ADAR KD dataset is the only dataset where all methods detected differential A-to-I editing. The distribution of LoDEI's $\delta^{A\to G}$ values are shown for windows exclusively detected by LoDEI (right) and for windows that overlap with sites detected by REDIT and/or JACUSA2 (left).

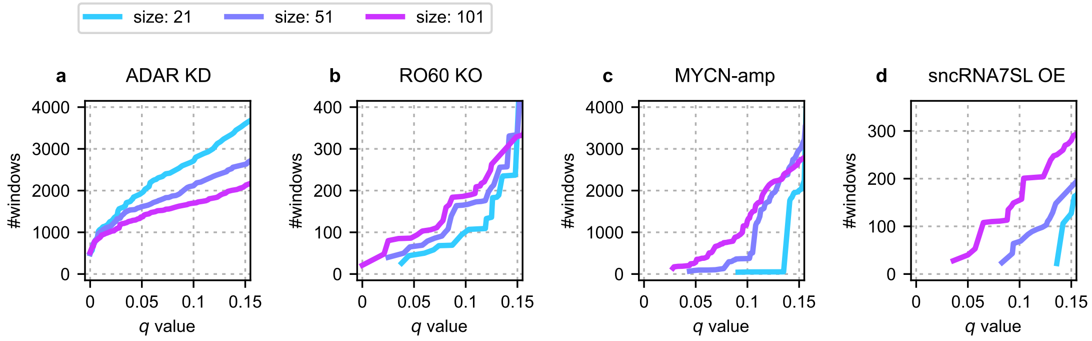

Supplementary Figure 7: Impact of the window size on the number of detected windows. The number of detected differential A-to-I windows as a function of the q value threshold is shown for the window sizes of 21nt, 51nt, and 101nt for the ADAR KD (a), RO60 KO (b), MYCN-amp (c) and *sncRNA7SL* (d) datasets.

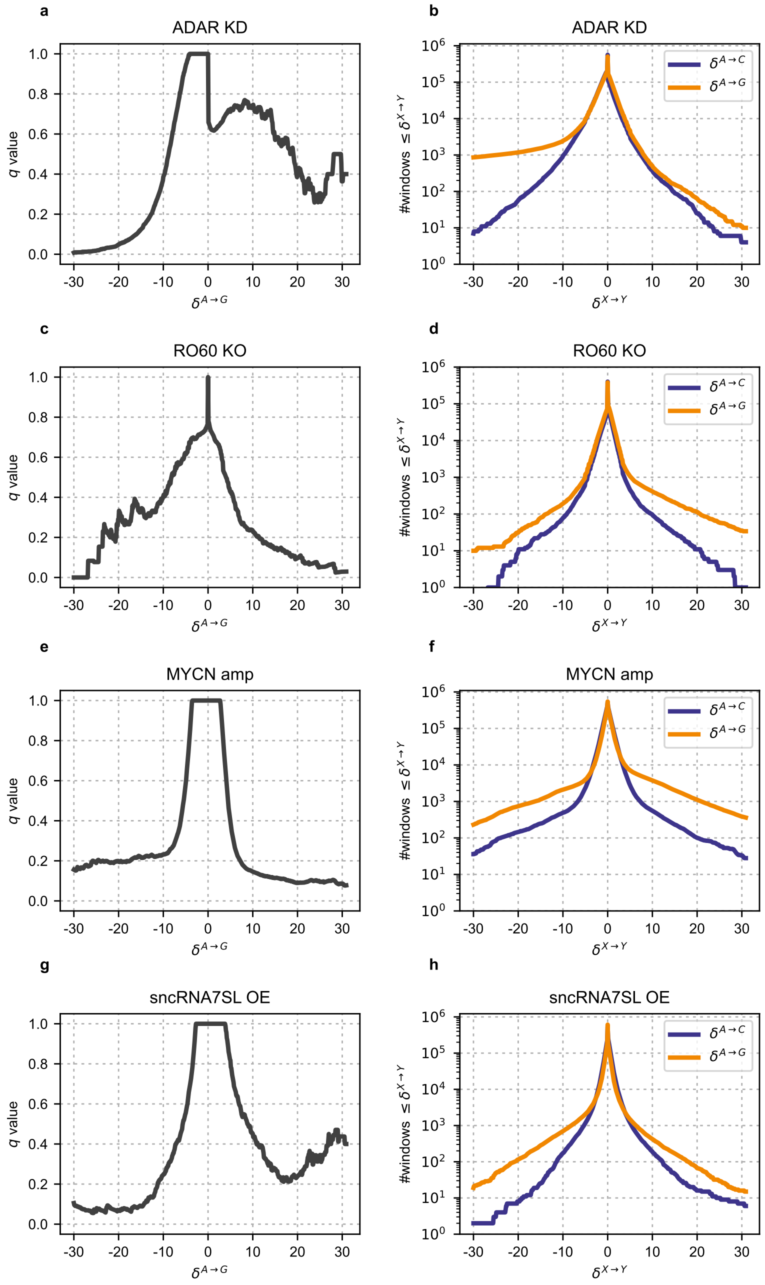

Supplementary Figure 8: Empirical q values and absolute number of detected windows. Empirical q values for $\delta^{A\to G}$ windows are shown based exclusively using $\delta^{A\to C}$ for the approximation of the number of false positives for the ADAR KD (a), RO60 KO (c), MYCN-amp (e) and *sncRNA7SL* (g) datasets. The absolute number of detected $\delta^{A\to G}$ and $\delta^{A\to C}$ are shown in (b), (d), (f), and (h). The stronger the δ signals, the less the number of available windows that can be used for the q value estimation causing a higher variance in the q value estimates. The higher variance can result in increasing q values against the overall trend of decreasing q values (e.g. q values in the RO60 KO dataset within the range of 15-30 of the $\delta^{A\to G}$ values).

a

chrom	wstart	wend	name	wEI	strand	q_value
chr1	225786901	225786951	**SRP9**	20.3	+	0.083

b Gene: SRP9 (signal recognition particle 9)
Position: chr1:225,786,912: silent mutation
Position: chr1:225,786,942 , silent mutation
Position: chr1:225786943, potential mutation of serine 75 (S75G)

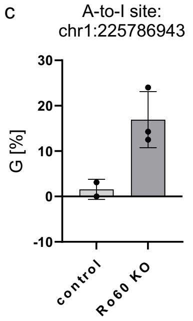

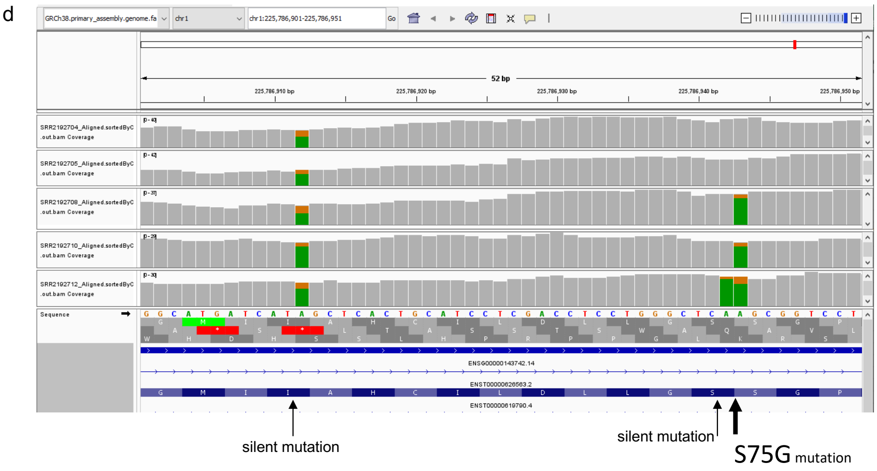

Supplementary Figure 9: Manual validation of differential A-to-I editing as detected by LoDEI in the RO60 KO dataset. a) LoDEI information, b) Differential edited sites, c) Percent differential A-to-I editing frequency. In control cells about 1.6% ($n = 2$) of sites are edited whereas in RO60 17% ($n = 3$) of the sites are edited. d) IGV browser screen-shot of a LoDEI window chr1:225,786,901-chr1:225,786,951. Three differentially edited sites are indicated (arrows). The bold arrow indicates a potential S75G mutation.

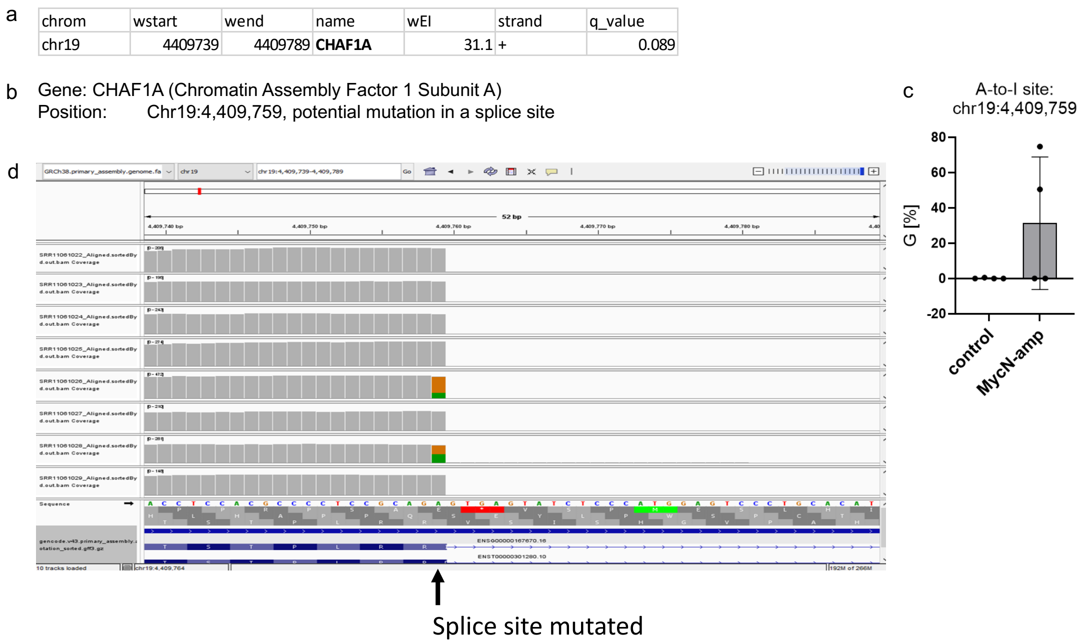

Supplementary Figure 10: Manual validation of differential A-to-I editing as detected by LoDEI in the MYCN dataset. a) LoDEI information, b) Differential edited sites, c) Average percent differential A-to-I editing frequency. In control cells about 0.6% ($n = 4$) of sites are edited whereas in MYCN-amp cells 31% ($n = 4$) of the sites are edited. d) IGV browser screen-shot of a LoDEI window chr19:4,409,739 – chr19:4,409,789 One differentially edited site is indicated. The bold arrow indicates the potential mutated splice site. This differential A-to-I editing site is not found in REDIportal.

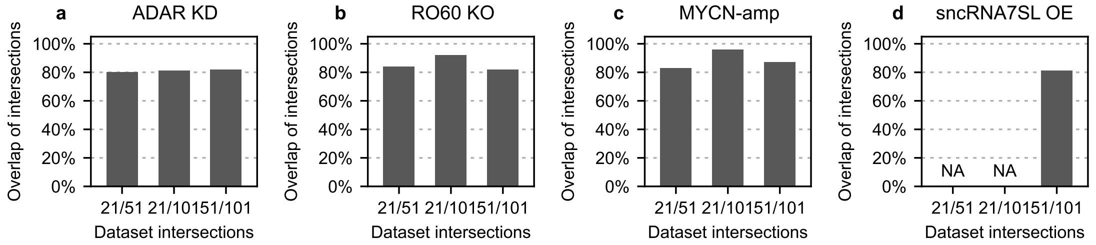

Supplementary Figure 11: Overlap of genomic positions of LoDEI windows with different window sizes. LoDEI was run with different window sizes (Supplementary Fig. 7). Here, the percent of overlap of the results of smaller windows with the results of larger windows for a q value threshold ≤ 0.1 are shown. Results obtained with a window size of 21 are compared against the results of window sizes of 51 (21/51) and 101 (21/101), and the results obtained with a window size of 51 are compared with results of the window size 101 (51/101). All overlaps are $\geq$ 80%.

1 C. elegans analysis

To further support the general applicability of LoDEI we performed the same analysis as in the main manuscript to previously published *C. elegans* data [1]. Wildtype *C. elegans* N2 RNA-seq data is compared against RNA-seq data from ADAR mutant strains in the embryo and L4 stage of the worm development. Similar to the findings in the human ADAR KD dataset, a strong contrast between $A \rightarrow G$ and non-$A \rightarrow G$ differences can be observed (Supplementary Fig. 12a vs. 12b and 12d vs. 12e; Fig. 2a vs. 2b). Non-$A \rightarrow G$ differences show a different pattern compared to the $A \rightarrow G$ signals. Strong δ values are exclusively detected in the $A \rightarrow G$ signals and do not appear in the background non-$A \rightarrow G$ signals, supporting the general applicability of LoDEI's approach to detect signals caused by A-to-I editing.

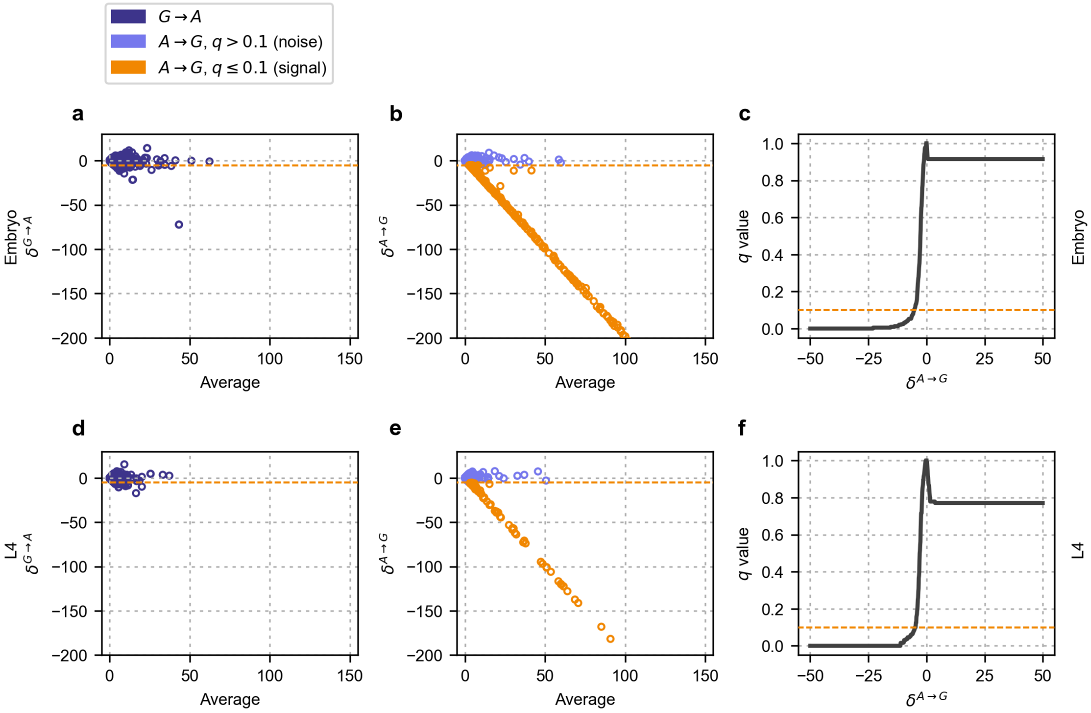

Supplementary Figure 12: Observed signal differences and empirically derived q values. Rows show the comparison of wildtype and ADAR mutant strains for the *C. elegans* embryo (a, b, c) and for the *C. elegans* L4 stage (d, e, f). The left column (a, d) shows Bland-Altman plots for $\delta^{G \rightarrow A}$ values representing the observed noise. The second column (b, e) shows Bland-Altman plots for $\delta^{A \rightarrow G}$ values which are a mixture of A-to-I editing signals and noise. Highlighted orange dots have an empirical q value ≤ 0.1. No strong δ values can be observed in the non-$A \rightarrow G$ comparison (left column) in contrast to the middle column. The right column shows empirical derived q values as a function of the δ signal.

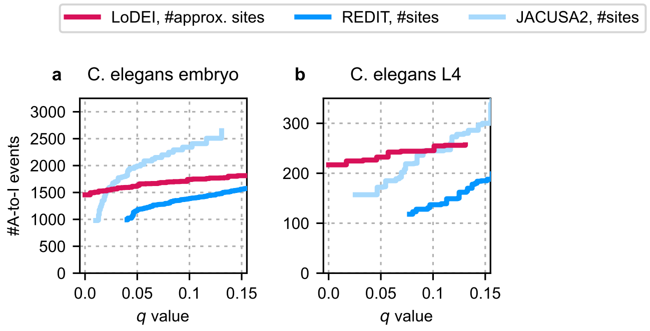

Supplementary Figure 13: Differential A-to-I site performance comparison in *C. elegans* datasets. The number of detected differential A-to-I sites is shown as a function of the q values threshold for the *C. elegans* embryo (a) and *C. elegans* L4 datasets (b).

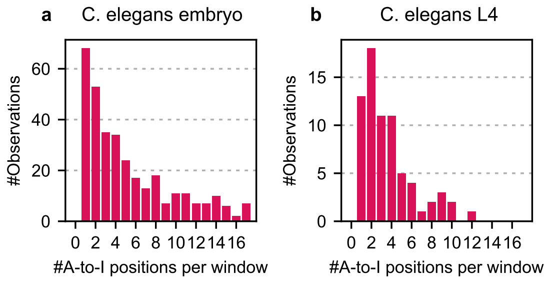

Supplementary Figure 14: Distributions of the average number of A-to-I positions per window in the *C. elegans* datasets. Shown are the average number of A-to-I sites within detected differential A-to-I windows by LoDEI in the *C. elegans* embryo (a) and *C. elegans* L4 datasets (b).

2 Comparison of single samples

To test if LoDEI might be able to detect differential A-to-I editing between single samples, we first generated results using LoDEI for each pairwise comparison between individual samples of sets S and S'. When naming individual samples only by their numeric index starting at 0, we can use the numeric indices of both samples to indicate which samples were used to generate the results. For instance, '01' is the name of the LoDEI result of the comparison of sample s_0 from set S with sample s_1 from set S' (Supplementary Fig. 15). We use this naming scheme in Supplementary Fig. 16.

To compare the detected differential A-to-I editing obtained from the pairwise comparisons, we used the Jaccard index. The Jaccard index $J(A, B)$ measures the similarity of two sets A and B by dividing the intersection of A and B by the union of A and B:

$$J(A, B) = \frac{A \cap B}{A \cup B} \tag{1}$$

Since LoDEI reports windows and not single positions, we first generated a list of all genomic positions covered by the reported windows which yields the sets A and B.

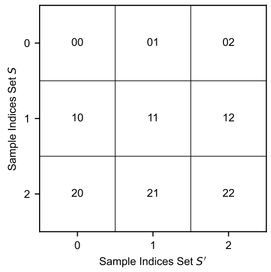

Supplementary Figure 15: Pairwise comparison naming scheme. To analyze the ability to detect differential A-to-I editing between single samples we first generated results using LoDEI for each pairwise comparison of the samples of the different sets. The entries in the matrix show the resulting names for a pairwise comparison. For instance, '01' is the name of the LoDEI result of the comparison of sample s_0 from set S with sample s_1 from set S'. This naming scheme is used in Supplementary Fig. 16

The generation of these lists is necessary to calculate a precise Jaccard index. Without transforming the windows into single positions, it would be questionable what an overlap between two windows is. For instance, an overlap of two windows by only a single position could be considered as an overlap of the results which could yield a larger overlap. To avoid such a potential bias and report a position-specific comparison of overlaps of windows, we first generate a list of the covered genomic positions and calculate the Jaccard index based on those genomic positions (Supplementary Fig. 16).

A detection of differential A-to-I editing detection based on the comparison of single samples could only be achieved in the ADAR KD and RO60 datasets where a strong difference in A-to-I editing is known.

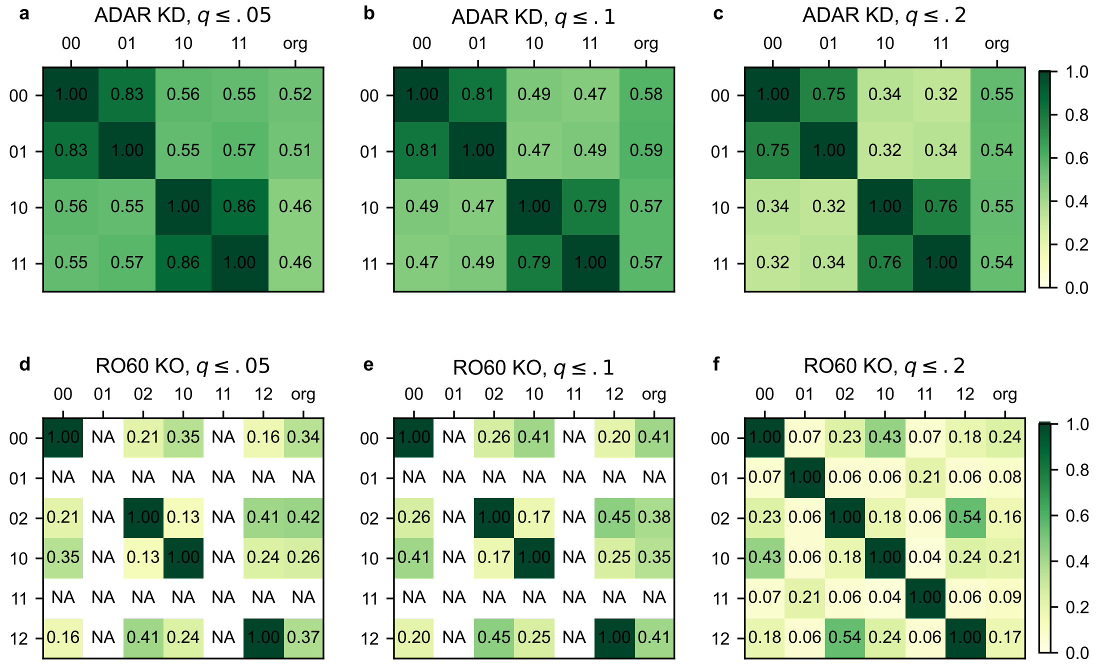

Supplementary Figure 16: Jaccard indices of detected differential A-to-I editing obtained from single sample comparisons by LoDEI. The Jaccard indices are shown between results from single sample comparisons. The 'org' column is the Jaccard index of a result of a single sample comparison with the result of the original result where the sets containing all samples were compared against each other. NA entries are caused if no windows with a q values ≤ 0.1 could be detected in the single sample comparisons.

3 Implications of a window-based differential A-to-I editing calculation

Three artificial scenarios are shown in Supplementary Fig. 17 a), b), and c), to help indicating the implications of window-based approaches in comparison to a site-specific detection approaches. In all scenarios, the samples s_1, s_2, s_3 belong to set S and the samples s_4, s_5, s_6 belong to set S'. Each scenario shows three adenosines within a single window. The shown adenosines are not required to be consecutive, but need to be anywhere within a window.

Scenario a) shows an example for a site-specific A-to-I editing event. Tools like REDIT and JACUSA2 were developed to detect this kind of signal. Since LoDEI first sums up the individual signals per sample in a window and then averages across these sums, individual editing events are also detected by the window-based approach used by LoDEI as shown by the intersection analysis in the results part of the main manuscript and Supplementary Fig. 5.

In scenario b), again one adenosine is edited per sample in set S like in scenario a), but here the editing takes place at different positions instead of the same position like

in scenario a). Site-specific tools like REDIT and JACUSA2 do not detect this scenario, since their statistical models require sufficient support of editing at the same position. In contrast, window-based approaches do not require a position specific editing and call a window being differential as long as there is a difference between the windows independent of the positions in the samples.

Since the differential editing is not position-specific in a window-based approach, no differential editing would be detected in scenario c) in a window-based approach. The overall editing per sample is identical for all samples. A position-specific approach would detect all 3 positions of being differentially edited, whereas position 1 and 3 would be stronger edited in S' and position 2 would be stronger edited in S.

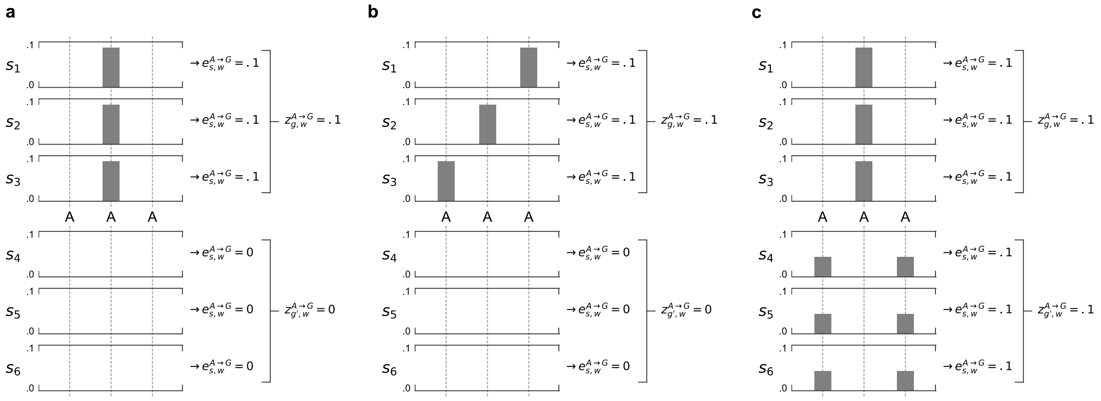

Supplementary Figure 17: Implications of a window-based differential A-to-I editing detection. Three artificial scenarios are shown to indicate the implications of a window-based detection approach. Samples $s_1, \ldots, s_3$ belong to set S and samples $s_4, \ldots, s_5$ belong to set S'. Scenario a) represents a site-specific editing event that can be detected by window-based and site-specific approaches. In scenario b) one adenosine is edited per sample in set S, but at different positions. Here, site-specific models do not detect differential editing in contrast to a window-based approach. In scenario c) window based approaches do not identify the shown positions as differentially edited, since the overall editing is identical per window. In contrast, site specific approaches would detect 3 differentially edited positions, whereas position 1 and 3 would be stronger edited in S' and position 2 would be stronger edited in S.

4 Supplementary Tables

Editing	Cutoff Negative	Cutoff Positive	LoDEI #-	LoDEI #+
ADAR1 KD				
AC	-inf	inf	0	0
AG	-11.1	inf	1624	0
AT	-inf	inf	0	0
CA	-inf	inf	0	0
CG	-inf	inf	0	0
CT	-inf	inf	0	0
GA	-inf	inf	0	0
GC	-inf	inf	0	0
GT	-inf	inf	0	0
TA	-inf	inf	0	0
TC	-21.5	inf	174	0
TG	-inf	inf	0	0
RO60 KO				
AC	-inf	inf	0	0
AG	-inf	16.6	0	114
AT	-inf	inf	0	0
CA	-inf	inf	0	0
CG	-inf	inf	0	0
CT	-inf	inf	0	0
GA	-inf	inf	0	0
GC	-inf	inf	0	0
GT	-inf	inf	0	0
TA	-inf	inf	0	0
TC	-inf	inf	0	0
TG	-inf	inf	0	0
MYCN-amp				
AC	-inf	inf	0	0
AG	-inf	30.4	0	271
AT	-inf	inf	0	0
CA	-inf	inf	0	0
CG	-inf	inf	0	0
CT	-inf	inf	0	0
GA	-48.2	inf	37	0
GC	-inf	inf	0	0
GT	-inf	inf	0	0
TA	-inf	inf	0	0
TC	-inf	inf	0	0
TG	-inf	inf	0	0
sncRNA7SL OE				
AC	-inf	inf	0	0
AG	-23.2	inf	64	0
AT	-inf	inf	0	0
CA	-inf	inf	0	0
CG	-inf	inf	0	0
CT	-inf	inf	0	0
GA	-inf	inf	0	0
GC	-21.3	25.6	108	76
GT	-inf	inf	0	0
TA	-inf	inf	0	0
TC	-inf	inf	0	0
TG	-inf	inf	0	0

Supplementary Table 1: LoDEI results for all possible mismatches for all analyzed datasets: Shown are the signal cutoffs corresponding to a q value ≤ 0.1 and the number of found windows for negative and positive $\delta^{A\rightarrow G}$ values. In cases of -inf or inf no signals with a q value ≤ 0.1 are found.

baseMean	log2FC	adj. p-value	gene
ADAR KD			
3388.72	-2.48	1.71×10^{-239}	ADAR
341.91	-0.90	4.68×10^{-8}	ADAR2
1.45	0.87	0.77	ADAR3
RO60 KO			
13800.97	0.08	0.81	ADAR
245.34	1.88	2.84×10^{-9}	ADAR2
232.35	1.15	0.27	ADAR3
MYCN-amp			
11135.97	-0.68	0.16	ADAR
1123.13	-1.93	0.009	ADAR2
27.85	2.17	0.43	ADAR3
sncRNA7SL			
17132.67	-0.06	0.23	ADAR
1365.72	-0.17	0.02	ADAR2
3.15	0.12	0.94	ADAR3

Supplementary Table 2: DESeq2 results for genes of the ADAR family: DESeq2 was run with default parameters testing for log2 fold changes being equal to zero. By default, DESeq2 adjusts derived p-values via the Benjamini-Hochberg method.

References

[1] Boaz Goldstein, Lily Agranat-Tamir, Dean Light, Orna Ben-Naim Zgayer, Alla Fishman, and Ayelet T. Lamm. A-to-I RNA editing promotes developmental stage-specific gene and lncRNA expression. *Genome Research*, 27(3):462–470, December 2016.

[2] Alexander Lex, Nils Gehlenborg, Hendrik Strobelt, Romain Vuillemot, and Hanspeter Pfister. UpSet: Visualization of Intersecting Sets. *IEEE Transactions on Visualization and Computer Graphics (InfoVis)*, 20(12):1983–1992, 2014.
